# Live Video Mind-Body Program for Patients With Knee Osteoarthritis, Comorbid Depression, and Obesity: Development and Feasibility Pilot Study

**DOI:** 10.2196/34654

**Published:** 2022-04-27

**Authors:** Ryan A Mace, Jonathan Greenberg, Nicole Lemaster, Brooke Duarte, Terence Penn, Millan Kanaya, James D Doorley, Jessica L Burris, Cale A Jacobs, Ana-Maria Vranceanu

**Affiliations:** 1 Integrated Brain Health Clinical and Research Program Center for Health Outcomes and Interdisciplinary Research, Department of Psychiatry, Massachusetts General Hospital Harvard Medical School Boston, MA United States; 2 Department of Orthopaedic Surgery & Sports Medicine, University of Kentucky Lexington, KY United States; 3 Integrated Brain Health Clinical and Research Program Center for Health Outcomes and Interdisciplinary Research, Department of Psychiatry, Massachusetts General Hospital Boston, MA United States; 4 Department of Psychology, University of Kentucky Lexington, KY United States

**Keywords:** knee osteoarthritis, depression, obesity, mind-body, physical activity, mixed-methods, mobile phone

## Abstract

**Background:**

Knee osteoarthritis (KOA) is the most common joint disorder in the United States and a leading cause of disability. Depression and obesity are highly comorbid with KOA and accelerate knee degeneration and disability through biopsychosocial mechanisms. Mind-body physical activity programs can engage biological, mechanical, and psychological mechanisms to improve outcomes in KOA, but such programs are not currently available.

**Objective:**

This mixed methods study aims to adapt a mind-body activity program for the unique needs of patients with KOA, depression, and obesity (GetActive-OA) delivered via live video.

**Methods:**

Participants were adults (aged ≥45 years) from rural Kentucky with obesity (BMI≥30 kg/m^2^), idiopathic KOA with mild to moderate radiographic changes, and elevated depressive symptoms (9-item Patient Health Questionnaire ≥10) recruited from 2 orthopedic centers. In phase 1, we developed GetActive-OA and the study protocol using qualitative focus group feedback from the study population (N=9; 2 focus groups, 90 minutes) and multidisciplinary expertise from clinical psychologists and orthopedic researchers. In phase 2, we explored the initial feasibility, credibility, and acceptability of GetActive-OA, live video delivery, and study procedures via an open pilot with exit interviews (N=5; 1 group). This research was guided by National Institutes of Health (NIH) model stage IA.

**Results:**

Phase 1 qualitative analyses revealed nuanced information about challenges with coping and increasing activity, high interest in a mind-body activity program, program participation facilitators (flexibility with technology) and barriers (amotivation and forgetfulness), and perceived challenges with data collection procedures (blood and urine samples and homework). Phase 2 quantitative analyses showed that GetActive-OA met most a priori feasibility markers: acceptability (80%), expectancy (100%), credibility (100%), clinician adherence (90%), homework adherence (80%), questionnaire data collection (100%), program satisfaction (100%), and safety (100%). Adherence to ActiGraph wear (80% baseline, 20% posttest) and collection of blood samples (60%) were low. Participation in GetActive-OA was associated with signals of improvements in general coping (Cohen *d*=2.41), pain catastrophizing (Cohen *d*=1.24), depression (Cohen *d*=0.88), anxiety (Cohen *d*=0.78), self-efficacy (Cohen *d*=0.73), pain (Cohen *d*=0.39), and KOA symptoms (Cohen *d*=0.36). Qualitative exit interviews confirmed quantitative findings and provided valuable information to optimize the program and protocol.

**Conclusions:**

Patients with KOA, depression, and obesity from rural Kentucky were interested in a live video mind-body activity program. GetActive-OA shows promise; however, the program and protocol require further NIH stage I refinement before formal efficacy testing (NIH model stage II).

**International Registered Report Identifier (IRRID):**

RR2-10.1016/j.conctc.2021.100720

## Introduction

### Background

Symptomatic knee osteoarthritis (KOA) is the most common joint disorder in the United States and is projected to affect >67 million Americans by 2030 [1,2]. Approximately one-third of patients with KOA experience rapid progression of cartilage degradation, knee pain, and disability [3], leading to greater health care use [4,5]. Depression and obesity, which are highly comorbid among patients with KOA [6-8], place individuals at a greater risk for these poor outcomes.

Depression, obesity, and KOA exacerbate one another through biopsychosocial pathways. Specifically, these conditions share a common pathophysiology [9-11] that involves a cycle of increased proinflammatory cytokine interleukin 1-beta (IL-1β) and Toll-like receptor 4 (TLR4) [3,12,13] activity, which, in turn, leads to inflammation-induced knee cartilage degradation [14]. KOA, depression, and obesity also exacerbate one another though a *disability spiral* that involves reduced physical activity [15], more pain, low mood, higher weight, and further knee cartilage degradation [16]. Current treatments (eg, medications, injections, and knee arthroscopy) are costly [17] and have limited efficacy [18], likely because they do not address the aforementioned biopsychosocial pathways that reinforce knee cartilage degradation, disability, and obesity. Novel treatments are needed to target the biopsychosocial processes involved in the KOA, depression, and obesity comorbidity.

Physical activity can improve depression, obesity, KOA pain, and cartilage breakdown [19]; however, uptake and adherence are challenging [20,21]. Indeed, there are barriers to engaging in physical activity in this population, including pain intensity, misinterpretation of pain signals as threats, and programs that are too challenging or incompatible with patients’ lives [22-25].

Walking is a promising, safe, and patient-preferred method for improving physical function in populations with chronic pain, particularly when it is gradually and strategically increased (ie, quota-based pacing regardless of symptoms) [22,26-32]. In addition, mind-body programs, which incorporate a range of complementary practices (eg, meditation, relaxation, breathing, and body movement) [33], can decrease depression, obesity, and pain in osteoarthritis (OA) [34-36], which are additional barriers to engagement in activity in this population. Combining mindfulness with walking is promising for improving mood and coping with KOA [35], and trials of this approach are ongoing (eg, NCT03064139). Multimodal programs that teach a variety of mind-body, walking, cognitive behavioral, and resiliency skills are needed to address depression, obesity, and pain as barriers to physical activity in OA and to target the biopsychosocial pathways of this comorbidity [37-42].

We have previously developed a multimodal, evidence-based program [43-46] for adults with chronic pain (GetActive) that combines mind-body skills with walking. GetActive has demonstrated high feasibility, acceptability, and satisfaction as well as statistically significant improvement in pain and physical and emotional function outcomes when delivered in person to adults with chronic pain [28,32]. Adapting this program for live video delivery would be an attractive option for patients with this comorbidity, including those living in rural areas. Depression, pain, and stigma associated with obesity can make weekly travel for clinic appointments challenging. Furthermore, live video delivery bypasses several barriers to care, including the lack of skilled providers in remote areas, missed work, and the burden of travel (cost and reliance on family and friends). This is particularly relevant for our patients in Kentucky with this comorbidity, who are underserved and prefer telehealth as a treatment modality. GetActive may be a solution to the problem of comorbid KOA, depression, and obesity, but it requires adaptations.

### Objectives

Here, we followed the National Institutes of Health (NIH) stage model [47] and conducted a mixed methods study aimed at adapting the original GetActive program for live video delivery and meeting the unique needs of patients with KOA, depression, and obesity (GetActive-OA) from rural Kentucky (NIH stage IA). In phase 1, we developed the live video GetActive-OA program and protocol through qualitative feedback from patient focus groups and multidisciplinary experts. In phase 2, we explored the initial feasibility, credibility, and acceptability of the program and preliminary signals of improvement in pain, multimodal physical function, emotional function, coping, and KOA biomarker outcomes. Individual exit interviews were then conducted to assist in further optimizing the programs and methodology before conducting a subsequent efficacy trial (NIH stage II).

## Methods

### Overview

Our methodology followed our previously published study protocol [48]. A total of 7 physicians at the University of Kentucky (UK) Healthcare Hip & Knee Center and the UK Healthcare Orthopedic & Sports Medicine Center referred eligible patients with mild to moderate KOA, using standard diagnostic criteria, during regularly scheduled office visits. The standard diagnostic criteria included clinical examination, patient-reported symptoms or functional limitations, and radiographic assessments. We also circulated an institutional review board–approved study flyer at the UK, Massachusetts General Hospital (MGH), and patient advocate groups such as the Arthritis Foundation.

### Ethics Approval

The institutional review boards at the UK and MGH approved all study procedures (approval number 53457 for the focus groups [Phase I] and 62256 for the open pilot [Phase II]).

### Participant Recruitment and Enrollment

Our eligibility criteria are consistent with those of other clinical trials in KOA or mind-body interventions [43,47]. The inclusion criteria were as follows: (1) obesity (BMI ≥30 kg/m^2^), (2) idiopathic KOA [49] with mild to moderate radiographic changes (Kellgren or Lawrence grade 2 or 3 [50]) or Knee Injury and Osteoarthritis Outcome Scores (KOOSs) consistent with KOA [51], (3) elevated depressive symptoms with a 9-item Patient Health Questionnaire (PHQ-9) score ≥10 [52,53], (4) aged ≥45 years [54,55], (5) history of concurrent psychotropics for <2 weeks before initiation of treatment or on stable doses for >6 weeks, (6) access to an internet-enabled computer or smartphone, (7) willingness to comply with the study protocol and assessments, and (8) physician’s clearance to participate. We selected the PHQ-9 because it is widely used and validated as a screener in health care settings [56], including orthopedic clinics [57], and for patients with KOA [58]. Given the tendency to underreport emotional symptoms of depression in our rural population, particularly among men, the PHQ-9 is also more likely to detect physical manifestations of depression that overlap with KOA (eg, slow movement and sleep disturbance).

The exclusion criteria were as follows: (1) any disorder requiring the use of systemic corticosteroids; (2) rheumatoid arthritis; (3) history of cancer within 5 years of screening; (4) inability to walk or use of a wheelchair; (5) previous surgical fixation of a femur or tibia fracture; (6) taking high doses of opioid pain medication (>50 mg of morphine equivalent per day); (7) diagnosis of a medical illness expected to worsen in the next 6 months (eg, malignancy); (8) active suicidal ideation or past-year psychiatric hospitalization; (9) non-English speaking; (10) lifetime history of schizophrenia, bipolar disorder, or other psychotic disorder; (11) current substance abuse or dependence (or a history within the past 6 months); (12) practice of yoga or meditation or other mind-body techniques once per week >45 minutes within the last 3 months; and (13) engagement in regular moderate or vigorous physical exercise for >30 minutes daily.

To reduce the travel burden for participants in rural Kentucky, we enrolled and collected baseline data (self-report questionnaires, blood draws, and urine samples) during the patient’s office visit with their referring physician whenever possible. After providing verbal consent, potential participants met with a trained research coordinator for study screening. Eligible participants provided written informed consent and Health Insurance Portability and Accountability Act authorization owing to the sensitive data collected. Trained clinical psychologists from MGH conducted the focus group and open pilot sessions via secure Health Insurance Portability and Accountability Act–compliant Zoom. After enrollment, the research coordinator either assisted participants with installing the Zoom app on their smartphone or emailed participants the Zoom installation instructions. The research coordinator also emailed the invitation link and appointment reminders to participants, offered Zoom test calls, and was available to solve technical difficulties in real time during the focus group and open pilot sessions.

### Phase 1: Development of GetActive-OA

The goal of phase 1 is to identify the treatment needs and preferences of patients with comorbid KOA, depression, and obesity from rural Kentucky via focus groups.

#### Focus Group Procedures

**Figure 1:**
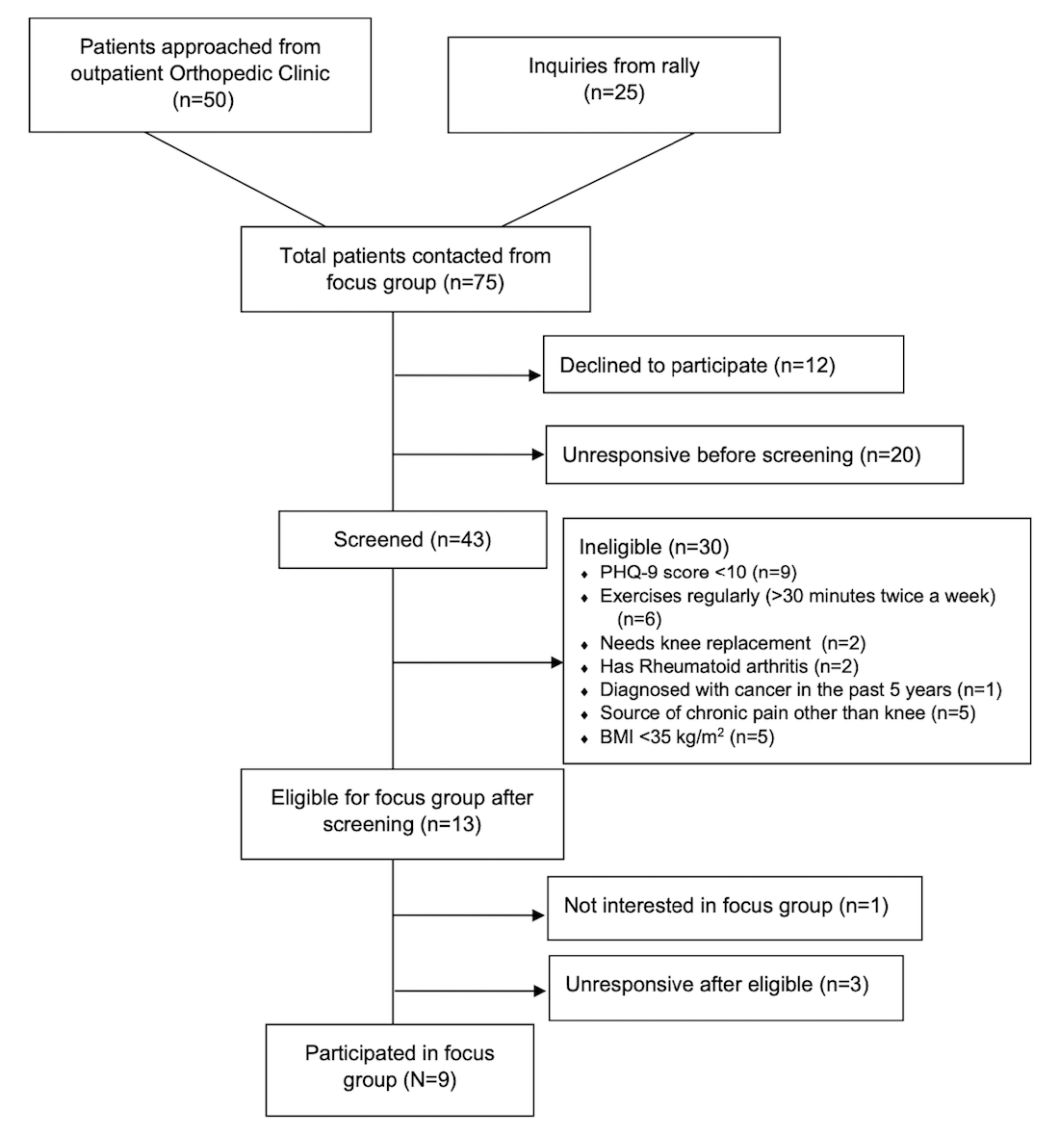
Participant flow for the focus groups (phase 1). PHQ-9: 9-item Patient Health Questionnaire.

Figure 1 depicts participant flow in phase 1.

Enrolled participants completed a one-time 90-minute focus group (N=9; a total of 4 groups). Clinical health psychologists with expertise in heterogeneous pain conditions and qualitative methods moderated the focus groups via Zoom. Our interdisciplinary team developed a semistructured qualitative interview guide to elicit feedback on the following a priori set themes: (1) experiences living with comorbid KOA, depression, and obesity (challenges, causes, and connections between conditions); (2) patients’ previous experiences with medical and complementary treatments; (3) perceptions of increasing walking; (4) perceptions of the GetActive-OA program, including barriers to program adherence (challenges with live-video delivery, group participation, homework completion); and (5) perceptions of data collection procedures (self-report assessments, ActiGraph, and blood and urine tests). The research coordinator transcribed the audio recordings of the focus groups.

We used rapid assessment procedures to analyze the focus group data, consistent with established qualitative frameworks [59-61]. Rapid assessment is a valid alternative to in-depth qualitative methods and is ideal for delivering timely results to guide multiple phases of intervention development [62,63]. We used rapid assessment to identify actionable suggestions to modify the GetActive-OA program and the study procedures for the phase 2 open pilot. First, the researchers created neutral domains that corresponded to each of the a priori set interview themes. Next, the moderators (RAM and JG) created a summary template to take notes during focus groups and reflexively summarize the main ideas directly afterward. The summary template was piloted with 1 focus group and modified, as necessary. The senior authors (CAJ and AMV) conducted a secondary review of the summaries to ensure consistency. In addition, the study team held regular meetings to collaboratively discuss any discrepancies in the summaries until a consensus was reached. Finally, we consolidated the summaries with a matrix to compare the focus groups, identified common themes, and reported the results in the next section [64].

#### Focus Group Results

Multimedia Appendix 1 reports detailed qualitative results by semistructured interview topic.

##### Theme 1: Experiences Living With Comorbid KOA, Depression, and Obesity

Participants attributed KOA, depression, and obesity to significant challenges across the domains of physical, emotional, and social functioning. Participants identified physical discomfort, pain flare-ups, and swelling as the primary sources of disability. Some participants were worried about deteriorating knee health, and a participant expressed further concerns that OA would negatively affect other parts of their body. Participants endorsed a variety of depressive symptoms, including low mood, amotivation, and feelings of hopelessness. Because of these emotional challenges, they described feeling socially isolated, lonely, stigmatized, embarrassed, and having low self-esteem. Most participants were knowledgeable about the range of psychological (eg, sedentary and dietary habits and life stressors) and biological factors (eg, heredity and aging) that contribute to KOA, depression, and obesity. However, some participants were unable to identify risk factors or endorsed misconceptions about KOA, such as activity causing knee flare-ups or being “too active” when young.

Participants described the co-occurrence of KOA, depression, and obesity as a “vicious cycle” of worsening disability. Many participants believed that depression led to unhealthy eating habits, subsequent weight gain, and knee pain. Other participants explained that KOA or pain flare-ups made it harder to move, resulting in weight gain and depression. Participants reported noticing themselves withdrawing from both physical and social activities and becoming more depressed over time. They reported frustration and discouragement by the lack of available or effective treatments.

##### Theme 2: Previous Experiences With Medical and Complementary Treatments

All participants reported a history of 1 or more medical treatments for KOA and knee pain including injections, surgery, pain medication, and steroids. The medical treatments were described as providing mixed results. Some participants reported that they had short-term relief, but the benefits wore off and they experienced negative side effects. Some participants were informed that they were ineligible for surgery because of their weight. One person reported having a *botched surgery*. Similarly, many participants reported prescriptions for antidepressant medication with modest benefit and negative side effects (eg, *brain fog*). Participants felt that their physicians commonly focused on 1 comorbidity and did not view them as a whole person.

Participants were knowledgeable about nonpharmacological interventions for pain (eg, physical therapy and mind-body activities) and depression (eg, psychotherapy and self-care) and weight loss interventions (eg, weight loss programs, personal trainers, and dietitians). Participants endorsed high interest and motivation in these approaches, as evidenced by initial lifestyle changes and help-seeking behaviors (eg, contacting physicians for referrals, removing junk food from pantry, and purchasing walking shoes). However, participants reported struggling to follow through because of lack of time, poor planning, distractibility, negative coping strategies (eg, withdrawal and avoidance, and overeating *comfort foods*), weather, and lack of insurance.

##### Theme 3: Perceptions of Increasing Walking

Overall, participants shared positive views of a program that encourages increased walking. Most identified walking as their preferred and primary source of physical activity. Some participants expressed an interest in walking more, whereas others reported that they already walk a lot for work. Participants recognized a bidirectional relationship between increasing walking and healthy eating or losing weight, which motivates them to continue positive habits (*building momentum*).

Participants identified several personal facilitators for walking, including setting gradual and feasible goals, pacing, creating reinforcements, and prioritization. Participants valued encouragement from others or having a *walking buddy*. Several participants used technology to promote walking, such as listening to music or using guided smartphone apps. Warm weather and having access to safe places to walk and exercise equipment were identified as environmental facilitators of physical activity.

Participants acknowledged several personal barriers to walking, including fear of further pain or injury, lack of motivation, procrastination, and time. A participant expressed concerns that walking is unsafe for patients with KOA and preferred to use a stationary bike. Several participants also identified a lack of access to safe walking areas (eg, few sidewalks and uneven surfaces) and COVID-19 restrictions as environmental barriers to walking. A participant noted that after overcoming these initial barriers, they were typically able to sustain their momentum and continue walking.

##### Theme 4: Perceptions of the GetActive-OA Intervention Components

Participants expressed a high interest in a program that combines increased walking with mind-body skills. Many participants agreed with the program’s rationale for addressing the 3 comorbidities. They believed that the mind-body skills would help them overcome the barriers to walking and healthy eating identified in theme 3. Participants also liked that the program did not include medication. Many participants noted the importance of balancing intervention components targeting KOA, obesity, and depression in every session. To reduce stigma surrounding obesity and depression, participants recommended the use of sensitive language in the treatment manuals and that the study clinicians speak about these issues in positive ways (“do not force dietary information”). Some participants expressed a fear of increasing walking because of the possibility of falling. Few participants had previous mind-body experience and were unsure if mindfulness would help but were open to trying it. Participants explained that reassurance from trusted sources, including medical clearance from their physicians or patient testimonials, would reduce their ambivalence about walking and mind-body skills.

With respect to format and delivery, participants were in support of participating in a live video group setting. Participants shared positive impressions of Zoom during the focus group, including that it was feasible, they enjoyed learning the features, and it enabled flexible attendance. Many participants were familiar with live video to stay connected with friends and family during the COVID-19 pandemic. Barriers to attending a live video group program included a lack of privacy at home, scheduling conflicts, and internet connectivity. Participants also noted several factors that would facilitate participation, such as support from the group and clinicians, regular reminders for walking and attendance, calendar appointments, and easy access to readings and program materials.

Participants also agreed that 5 to 10 minutes of home practice per day was feasible and constructive. They believed that home practice could encourage them to track progress in their walking goals and mind-body practice. Participants reported that a smartphone log would be easier and faster than paper and pencil. A participant noted that they might need assistance with using the smartphone log. Additional barriers to home practice include distractions, low motivation, forgetfulness, and schedule conflicts. A combination of reinforcements, such as reminders to log their home practice, check-ins from clinicians, and identifying family support, could prevent nonadherence.

##### Theme 5: Perceptions of Data Collection Procedures

There were no major concerns regarding the biological data collection of blood and urine samples. A few participants noted previous experience with phlebotomists who had difficulty locating a vein for blood collection. Several participants noted the inconvenience of traveling to the clinic, including long commutes, transportation costs, and difficulty finding parking. Nevertheless, participants confirmed that the biological data collection could not deter them from participating. Participants recommended combining the biological data collection with physician appointments to reduce this burden. Many participants also expressed an interest in receiving the results to learn about their inflammation levels and other markers of knee health.

Similarly, most participants had no concerns about ActiGraph or self-report assessments. Participants expressed a preference for a small, unobtrusive, and well-fitting device. A participant was concerned about privacy and wanted more information on whether ActiGraph could track the geographical location. Several participants had experience with self-reports for research or medical appointments and preferred to complete them via a web-based survey. A participant noted the importance of explaining the purpose of the self-report assessments so that they “don’t feel like an exam.”

### Phase 2: Preliminary Feasibility of the GetActive-OA Program

#### Overview

The goal of phase 2 is to conduct an open pilot study of the newly developed GetActive-OA with individual exit interviews to explore preliminary credibility, acceptability, satisfaction with treatment, feasibility of recruitment, instruments, biological data collection, and adherence to homework (exercise and mind-body skills). Here, we describe modifications to the GetActive-OA program and the phase 2 procedures informed by our phase 1 qualitative focus group results. Our published protocol [48] contains remaining details of procedures that were not influenced by the focus groups, including clinician adherence, depression severity and suicide risk assessment, and the GetActive-OA makeup session.

#### GetActive-OA Program and Procedures

Table 1 presents the program outline and skills for each session.

GetActive-OA also provides educational information on the biopsychosocial interactions among KOA, depression, and obesity that comprise a population-specific *disability spiral*. Participants learn that inflammation is the biological tie among the 3 conditions and physical activity is a modifiable factor that can decrease inflammation, improve the biology and function of the knee, and decrease both depression and obesity [7-9,16,38-40,65-67]. GetActive-OA teaches 5 core skills that target the comorbidities: (1) setting weekly activity goals that are personally meaningful (eg, walking with kids instead of forcing a gym workout); (2) quota-based pacing to gradually and safely increase activity that is noncontingent on pain (eg, walking for 15 minutes twice per day); (3) mind-body skills to elicit relaxation and cultivate mindfulness (eg, deep breathing and mindfulness meditation) and minimize negative reactivity to pain and reduce activity avoidance; (4) cognitive behavioral skills (eg, behavioral activation and adaptive thinking) to challenge pain-specific cognitions such as catastrophizing and fear avoidance that interfere with program goals; and (5) resiliency skills including self-compassion, gratitude, acceptance, and social support to enhance coping, given that discouragement is common in this population.

We also added several novel components based on the focus group results. First, we added patient-friendly educational information on the interconnectedness of KOA, depression, and obesity (eg, reduced activity can exacerbate pain and disability). Second, participants were encouraged to apply mindfulness to facilitate healthy eating and dietary changes (eg, noticing hunger or fullness urges and mindful eating). Third, our multidisciplinary team reviewed all skills for patient-sensitive language and to reduce stigma. Fourth, we provided all participants with a stationary peddler. This was intended to serve as an alternative to walking for participants with concerns about starting a walking program or with limited access to sidewalks in rural Kentucky. It may also address other environmental barriers to walking, such as bad weather, as identified in the focus groups or participants who live in neighborhoods with high crime rates.

Finally, given the positive impressions of technology and to reach patients in rural areas, we optimized the program and study procedures for live video delivery. We have successfully adapted mind-body programs for live video similar populations with chronic pain [45,68]. We considered other approaches to live video, such as asynchronous web platforms, but decided against it because telehealth delivery is preferred by this population and fostering peer contact can directly target stigma and isolation common in this comorbidity. To offset the weaknesses of live video groups (eg, scheduling), we created a website that contains program materials and audio recordings of mind-body skills that participants can access at any time.

A clinical psychologist from MGH led the 8 weekly GetActive-OA group sessions (90 minutes) via Zoom with the support of a clinical psychology fellow. The first session of GetActive-OA oriented participants to program expectations for participation. In each weekly session, the study clinician introduced the core GetActive-OA skills and problem-solved adherence issues. Participants’ home practice involved daily walking or stationary pedaling according to their activity goal, daily mind-body skills (5-10 minutes, via clinician-guided audio recordings available on the program website), and logging in the GetActive-OA manual at least three examples of gratitude. Participants who achieved their activity goal from the previous week were encouraged to increase their goal by 10% to 20%, according to the guidelines for quota-based pacing [69]. Participants also logged their mindfulness minutes and skills practiced each day using a smartphone log. The research coordinator sent reminders (via SMS text messaging, phone call, or email based on participant preference) to remind participants of the homework and sessions.

**Table 1:** GetActive-OA session overview informed by the focus group results.

Topic	GetActive-OA skills
1. Break the disability spiral by exercising	Myths about pain, disability spiral, and quota-based pacing
2. Smart ways to exercise more	Exercising with enjoyment, self-compassion and gratitude, and diaphragmatic breathing
3. Mindfulness	Mindfulness, mindful breathing, body scan, and mindful moments
4. Everyday mindfulness	Leaning into difficulty, mindfulness of pain, mindful exercising, and noticing the benefits of exercising
5. The benefits and barriers to exercise	Mindful eating, overcoming barriers to exercising, stop and breathe, reflect, and choose
6. Coping with negative thoughts	Negative automatic thoughts, changing our perspective, and acceptance
7. Strengthening social support	Social support and the disability cycle, effective communication, social walking, and loving kindness
8. Staying on track and maintaining your progress	The powerful self; working with pain, your emotional well-being, and unhealthy weight; and resiliency plan

#### Measures

##### Feasibility Markers

We evaluated a priori appropriateness, acceptability, feasibility, and fidelity based on established benchmarks, consistent with our prior feasibility pilot studies [31,32,70] and protocol for GetActive-OA [48].

- Program credibility and expectancy were determined by the percentage of participants with Credibility and Expectancy Questionnaire–6 [71] scores above the scale’s midpoint.
- Program satisfaction was determined by the percentage of participants with Client Satisfaction Questionnaire–3 [72] scores above the scale’s midpoint.
- Feasibility of recruitment was determined by the percentage of patients who agreed to participate from the total patients approached.
- Program acceptability was determined by the percentage of patients who attended at least six of the eight of the GetActive-OA sessions.
- Adherence to ActiGraph wGT3X-BTLink was determined by the percentage of participants with valid accelerometer data for at least five of seven days for a minimum of 10 hours per day during the baseline and postintervention testing.
- Adherence to the home practice was determined by the percentage of participants who completed mind-body and walking skills at least four of seven days or one of these skills at least five of seven days.
- Feasibility of assessments was determined by the percentage of participants with no missing outcome data.
- Study clinician adherence was determined by the percentage of content delivered based on an independent audit of audio recordings and progress notes for all sessions by the senior authors.
- We assessed program safety based on the absence of adverse events (eg, swelling soreness and stiffness) and stable medication use reported to the study staff.

##### Quantitative Assessments

Our assessments aligned with the Initiative on Methods, Measurement, and Pain Assessment in Clinical Trials, Osteoarthritis Research Society International, and Outcome Measures in Rheumatology recommendations [73,74]; our conceptual model [48]; and recommendations for feasibility trials [75,76]. In addition, 1 week before and after the GetActive-OA program, participants traveled to the UK to receive their ActiGraph activity monitor (model wGT3X-BTLink) and collect biological data. Participants completed the self-report assessments on the web via REDCap (Research Electronic Data Capture; Vanderbilt University).

The Numerical Rating Scale (NRS) [77] assessed pain intensity at rest and with activity on an 11-point scale. Higher scores indicated greater severity of pain (0, no pain; 10, worst pain). The minimum clinically important difference (MCID) for the NRS was 1 [78].

The KOOS [79] assessed KOA-specific pain (9 items), KOA symptoms (7 items), activities of daily living (17 items), sport and recreation function (5 items), and knee-related quality of life (4 items). Items were scored on a scale from none (0) to extreme (4) or never (0) to always (4). Subscale scores were transformed to a scale of 0 to 100, with lower scores indicating more severe knee problems. Of the 5 subscales, 4 (80%) were used in this study’s analyses, as the Sport and Recreation Score was not applicable to this population, and MCIDs for the 4 subscales were as follows: pain=9.3, symptoms=8.4, activities of daily living=9.0, and quality of life=10.3 [80].

The ActiGraph wGT3X-BTLink accelerometer assessed the average step count over a 7-day period at the baseline and postintervention time points. Our published protocol contains full details of the accelerometer procedures [48]. Participants had to wear ActiGraph on their nondominant wrist for a minimum of 10 hours per day for a minimum of 5 days for their data to be considered valid during the baseline and postintervention testing [32,48]. Participants did not wear the ActiGraph wGT3X-BTLink during the program and did not have access to their step count data, as the device itself does not have a display screen. We opted to use the *blinded* ActiGraph so that any change in physical activity would not be confounded by the feedback provided by the device. We processed the accelerometer data using the ActiLife software [81] according to established guidelines [82-84]. The MCID for the accelerometer was 800 steps/day, which is consistent with that used in other clinical populations [85].

The Physical Activity Scale for Individuals With Physical Disabilities (PASIPD) [86] assessed self-report of disability on a 13-item scale. Lower total scores, calculated based on the average hours per day and metabolic equivalent values, indicated higher disability across leisure, household, and work-related activities. The MCID for the PASIPD is not available.

The Patient-Reported Outcomes Measurement Information System (PROMIS) Anxiety (v1.08a) and PROMIS Depression (v1.08b) [87] both assessed emotional functioning on separate 8-item scales. Participants were asked about the frequency of their anxiety and depression symptoms (1, never; 5, always). Higher *T* scores indicated greater severity of anxiety and depression. The MCID was 4.28 for the PROMIS Anxiety and 5.19 for the PROMIS Depression [88].

The Pain Catastrophizing Scale (PCS) [33] assessed hopelessness, helplessness, and negative rumination about pain on a 13-item scale. Higher scores (range 0-52) indicated greater pain catastrophizing (0, not at all; 4, all the time). An MCID was not available for the PCS.

The Arthritis Self-Efficacy Scale (ASES) [89,90] assessed arthritis-specific self-efficacy on a 20-item scale. The ASES contains pain (5 items), function (9 items), and other symptoms (6 items) that are scored on a scale from very uncertain (1) to very certain (10). Scores ranged from 1 to 10, with higher average scores indicating greater arthritis-specific self-efficacy. The MCID for the ASES was not available.

The Measure of Current Status–Part A (MOCS-A) [91] assessed general coping skills on a 13-item scale. Participants rated their ability to use relaxation, awareness of tension, ability to express needs, confidence in coping, and assertiveness skills on a scale from 0 (I cannot do this at all) to 4 (I can do this extremely well). Higher total scores (range 0-52) indicated greater use of coping skills. An MCID was not available for the MOCS-A.

The Modified Patient Global Impression of Change (MPGIC) [92] assessed patients’ perception of improvement using the scale from 1 (very much improved) to 7 (very much worse) on 5 questions related to the following: pain, level of physical activity, physical function, emotional function, and resiliency.

We assessed KOA biomarkers of cartilage breakdown and systemic inflammation. Urinary CTXII (Urine Cartilaps, CTX-II; Immunodiangostic Systems Inc) was used to assess cartilage degradation, which was normalized to creatinine levels to account for differences in hydration [93] (Parameter Creatinine Assay; R&D Systems Inc). Enzyme-linked immunosorbent assays were used to assess the proinflammatory cytokines IL-1β and interleukin 6 (IL-6; Proinflammatory Multiplex 1; Meso Scale Diagnostics) as well as TLR4 (Invitrogen Human TLR4, Life Technologies Corporation). The selected biomarkers have been shown to predict inferior clinical outcomes and cartilage thinning [93,94] and are responsive to changes over a 3-month follow-up [95-97]. Our published protocol contains full details of the biological data collection [48].

##### Quantitative Analysis

This open pilot study was designed in accordance with the NIH stage model intervention development pilot studies to explore feasibility and *not* to test efficacy [47,98,99]. We reported the frequency and percentage of each a priori benchmark set in our previously published protocol [46]. We rated each benchmark as *good* if the criteria were met in at least ≥80% (4/5) of the open pilot study participants. The benchmarks allowed us to evaluate readiness for a subsequent efficacy trial and determine whether further modifications to GetActive-OA and study procedures are needed. Exploratory analysis for each quantitative measure included descriptive statistics, baseline and postintervention comparisons using paired 2-tailed *t* tests, and Cohen *d* effect sizes (0.20, small; 0.50, medium; and 0.80, large; [100]) to cautiously explore preliminary before and after improvement associated with participation in GetActive-OA.

##### Exit Interviews

We conducted group exit interviews via Zoom using the same rapid assessment procedures as in phase 1. Our goal is to gather impressions about the GetActive-OA adaptations and technology enhancements. We performed rapid assessment procedures on the exit interview transcripts to identify actionable suggestions to optimize GetActive-OA for the subsequent randomized controlled trial (RCT) [59]. Integrating the qualitative exit interviews with the quantitative results also allowed us to corroborate the feasibility markers, contextualize the findings at both the group and individual participant levels, and begin to understand *why* changes in outcomes may have occurred [101].

## Results

### Participants and Feasibility Markers

**Figure 2:**
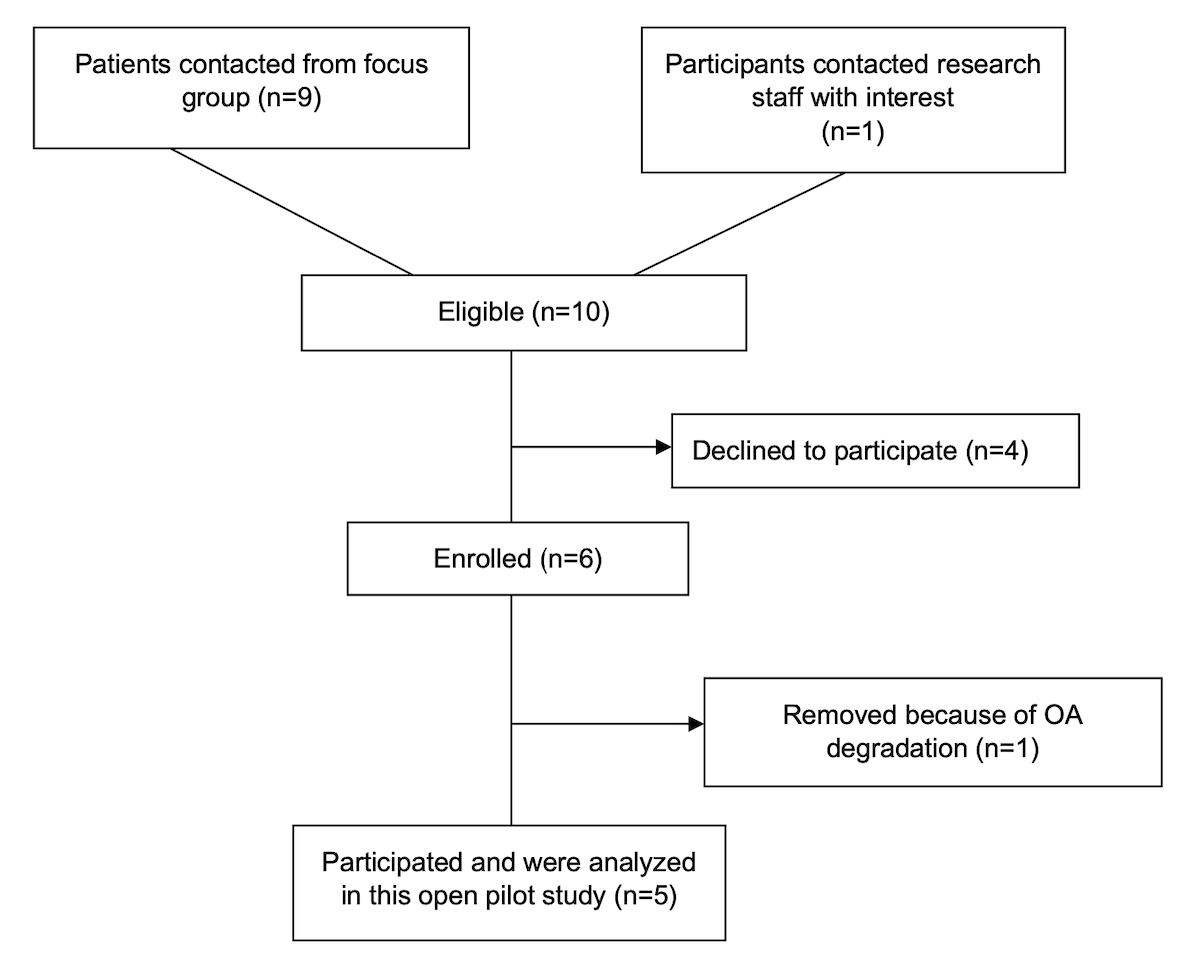
Participant flow for the live video open pilot study (phase 2). OA: osteoarthritis.

Figure 2 shows the flow diagram, and Table 2 reports the demographics of the open pilot study participants.

GetActive-OA had *good* feasibility on nearly all of the a priori benchmarks (Table 3) [31,32,48,70].

Of the 10 patients who were approached, 9 (90%) were eligible after screening. Of these 10 participants, 6 (60%) were enrolled after 4 (40%) declined to participate owing to disinterest in the study and lack of time (*poor feasibility of recruitment*). All but one of the enrolled participants, who was removed because of neurological symptoms that were not present at the time of enrollment, completed baseline testing and started the open pilot. The sample was majority White, non-Hispanic, women, married, and fully employed. More than half of the participants were prescribed psychotropic medication for depression or anxiety (3/5, 60%) and had bilateral KOA (3/5, 60%).

GetActive-OA was viewed as highly credible, and participants expected their knee health to improve during the program (5/5, 100% above the Credibility and Expectancy Questionnaire–6 scale midpoint, *good program credibility and expectancy*). Of the 5 participants, 4 (80%) attended at least six GetActive-OA sessions (*good program acceptability*), and 4 (80%) completed the smartphone homework logs (*good adherence to homework*). The study clinicians delivered 90% of the manual content (*good study clinician adherence*). Participants completed all self-report measures at the baseline and postintervention assessment time points (no missing data, ie, *good feasibility of quantitative measures*). At baseline, of the 5 participants, 4 (80%) met the adherence benchmark of a minimum of 10 hours of ActiGraph wear time for a minimum of 5 days (*good ActiGraph adherence*). However, of the 5 participants, only 1 (20%) met this benchmark at the postintervention time point, with 1 (20%) patient not meeting the minimum wear time for even 1 day (*poor ActiGraph adherence*). All participants were highly satisfied with the program (5/5, 100% above the Client Satisfaction Questionnaire–3 scale midpoint, *good satisfaction*). No adverse events were reported (*good program safety*).

**Table 2:** Demographics and clinical characteristics of open pilot study participants (N=5).

Characteristics			Value
Age (years), mean (SD; range)			53.2 (6.64; 49-65)
BMI (kg/m^2^), mean (SD; range)			39.8 (6.68; 32.6-50.8)
**Sex, n (%)**			
	Male	0 (0)	
	Female	5 (100)	
**Ethnicity, n (%)**			
	Hispanic or Latinx	1 (20)	
	Not Hispanic or Latinx	4 (80)	
**Race, n (%)**			
	White	4 (80)	
	African American	1 (20)	
**Marital status, n (%)**			
	Single, never married	1 (20)	
	Widowed	1 (20)	
	Married	3 (60)	
**Education, n (%)**			
	Completed high school or GED^a^	2 (40)	
	Associate’s degree	2 (40)	
	Some college	1 (20)	
**Employment, n (%)**			
	Employed full-time	3 (60)	
	Homemaker	1 (20)	
	On disability	1 (20)	
**OA^b^ symmetry, n (%)**			
	Bilateral	3 (60)	
	Unilateral	2 (40)	
**Depression or anxiety medication, n (%)**			
	Yes	3 (60)	
	No	2 (40)	
**Opioid medication, n (%)**			
	Yes	1 (20)	
	No	4 (80)	

^a^GED: graduate equivalency degree.

^b^OA: osteoarthritis.

**Table 3:** A priori feasibility markers.

Marker	Criteria
Feasibility of recruitment	Of 10 eligible patients, 6 (60%) successfully contacted agreed to participate (poor)
Program acceptability	Of 5 participants, 4 (80%) attended ≥63% (≥5/8) of group or make-up sessions (good)
Credibility and expectancy	Of 5 participants, 5 (100%) scored above the scale midpoint for expectancy (good), and 5 (100%) scored above the scale midpoint for credibility (good)
Study clinician adherence	The study clinicians delivered 90% of the manual content across the 8 sessions (good)
Feasibility of quantitative measures	Of 5 participants, 5 (100%) were not fully missing questionnaires on quantitative measures at baseline (good), and 5 (100%) were not fully missing questionnaires on quantitative measures at the postintervention time point (good)
Adherence to homework	Of 5 participants, 4 (80%) completed mind-body and walking skills at least four of seven days or one of these skills at least five of seven days (good)
Adherence to ActiGraph	Of 5 participants, 4 (80%) who received ActiGraph at baseline wore it for ≥5 of 7 days (good), and 1 (20%) who received ActiGraph at the postintervention time point wore it for 5 of 7 days (poor)
Client satisfaction	Of 5 participants, 5 (100%) scored above the scale midpoint (good)
Program safety and adverse events	0 adverse events

### Quantitative Outcomes

Table 4 reports the results for the quantitative outcomes.

Baseline ActiGraph step count varied widely (mean 8832, SD 4083; range 5548-16,410). Participants endorsed moderate to high levels of pain intensity (mean 7.0, SD 1.5) [102]. The levels of pain catastrophizing were higher than the norms for chronic pain samples (mean 39, SD 12.5). Participants had more severe knee problems than previous samples with OA across all KOOS measures [103]. Participants reported elevated levels (PROMIS+1 SD) of both depression (mean 60.6, SD 7.5) and anxiety (mean 60.8, SD 9.0).

As a group, participants exhibited small to moderate improvements in KOA pain, KOA symptoms, and KOA physical function on the KOOS. Participants reported large improvements in general coping on the MOCS-A, arthritis self-efficacy on the ASES, and reductions in pain catastrophizing on the PCS. Participants also showed large reductions in depression and anxiety on the PROMIS. Changes in KOA-related quality of life on the KOOS, pain intensity on the NRS, and disability on the PASIPD were minimal. Among the 4 patients with pre- and posttest ActiGraph data, there was a small decrease in the average step count. The 1 participant who met the adherence benchmark at the postintervention time point increased by 310 steps. Only 1 participant reported that their emotional functioning had improved on the MPGIC. Participants did not report improvements on the remaining MPGIC items: pain, physical function, and resiliency.

**Table 4:** Quantitative outcomes.

Measure	Baseline, mean (SD)	Postintervention, mean (SD)	Mean difference from the paired sample *t* test (2-tailed)	*P* value	Cohen *d*
KOOS^a^ pain	33.6 (5.5)	38.0 (14.9)	4.4	.36	0.39
KOOS symptoms	32 (16.8)	39.4 (22.8)	7.4	.18	0.36
KOOS ADL^b^	34.4 (6.7)	39.6 (14.8)	5.2	.54	0.45
KOOS QOL^c^	14 (6.8)	14 (15.7)	0	.99	0
NRS^d^ pain	7.0 (1.5)	7.2 (1.4)	0.2	.82	0.13
PASIPD^e^	36 (19.3)	37.6 (33.8)	1.6	.93	0.05
PROMIS^f^ Depression	60.6 (7.5)	52.6 (10.4)	−8.0	.08	0.88
PROMIS Anxiety	60.8 (9.0)	52.6 (11.7)	−8.2	.11	0.78
PCS^g^	39 (12.5)	24.4 (10.9)	−14.6	.10	1.24
ASES^h^	3.8 (0.8)	4.8 (1.7)	1.0	.30	0.73
MOCS-A^i^	22.8 (3.7)	33 (4.7)	10.2	.004	2.41
Step count^j^	8763 (3813.9)	7887 (3131.9)	−766	.37	0.22
IL-6^k^	3.10 (0.48)	1.97 (1.75)	−1.13	.38	0.87
TLR4^l^	24.27 (33.49)	16.58 (10.12)	−7.69	.74	0.34
CTX-II^m^	391.1 (153.1)	629.2 (404.3)	238.1	.25	0.76

^a^KOOS: Knee Injury and Osteoarthritis Outcome Score.

^b^ADL: activity of daily living.

^c^QOL: quality of life.

^d^NRS: Numerical Rating Scale.

^e^PASIPD: Physical Activity Scale for Persons With Physical Disability.

^f^PROMIS: Patient-Reported Outcomes Measurement Information System.

^g^PCS: Pain Catastrophizing Scale.

^h^ASES: Arthritis Self-Efficacy Scale.

^i^MOCS-A: Measures of Current Status–Part A.

^j^ActiGraph step count, which is the weekly average of the valid days (≥10 hours).

^k^IL-6: interleukin-6.

^l^TLR4: Toll-like receptor 4.

^m^CTX-II: Urine Cartilaps (Immunodiangostic Systems Inc).

### Biomarker Analyses

Urine samples were successfully collected from all patients at both pre- and posttest time points, and CTX-II and creatinine values were above the lower limits of detection for all samples. CTX-II, expressed as nanogram per millimole creatinine, moderately increased. Whereas urine samples were successfully collected, only 60% (6/10) of the total possible blood draws were successful because of low vein patency, thereby prohibiting meaningful analysis of pre- to posttest changes. From the available samples, all TLR4 and IL-6 values were above the lower limits of detection; however, 67% (4/6) of the samples had IL-1β levels below the limits of detection.

### Exit Interview Analysis

Participants shared positive overall impressions of the program, including the group setting, mind-body and activity skills, and home practice logs. Participants were more engaged with the program website than with the physical treatment manual. Almost all participants enjoyed the group setting. Several participants noted that the group normalized the challenges of living with KOA, unhealthy weight, and a low mood. The weekly meetings also increased motivation and accountability for their goals. Zoom was well liked, accessible, and feasible for everyone. Some emphasized the need to foster connections among group members (eg, exchange helpful information) to overcome the tendency for remote participation to be impersonal. There was some disagreement about program length; many reported that the 8 weeks and 90-minute sessions were too long, whereas a few were satisfied with these commitments. Other barriers to participation included previous commitments, life events, and health concerns. Reminders and individualized support from the study staff were helpful for staying engaged with the program.

Many reported improvements in their physical activity, pain-specific cognitions, and mood associated with the program components. Most notably, all participants highlighted mind-body skills as their favorite aspect of the program. Participants enjoyed the in-session mindfulness exercises and reported using mind-body skills outside of the session, with recordings on the program website. Participants described the mindfulness exercises as helpful when experiencing high levels of pain and for altering pain-specific cognitions (eg, use of deep breathing at night to dull throbbing sensations in the lower limbs, to manage pain, and to lessen rumination on pain experiences). Participants also reported positive impressions of the activity skills and that they increased their activity with pacing, individualized goal setting, and linking exercise to personally meaningful activities. A participant noticed that combining mind-body and activity skills, such as mindful walking, made gradual increases in activity safer. However, a few participants struggled to implement activity skills, citing lack of time and the ability to structure activity blocks into their daily routine.

Participants had mixed experiences of gratitude and self-compassion. Many participants acknowledged that these skills did not reduce self-criticism and self-doubt, which are both common in pain and depression. However, a participant noted that self-compassion resonated and helped them “break the spiral” of disability. Some participants believed that the healthy eating skills were helpful in changing dietary choices, whereas others believed that the information was redundant. Finally, adaptive thinking skills were generally not impactful on participants. Many did not recall these skills or implement them. However, a participant noted that adaptive thinking helped change their relationship with pain in a positive way.

## Discussion

### Principal Findings

Depression and obesity are highly comorbid with KOA, can accelerate knee degeneration, and share a common pathophysiology involving systemic inflammation and proinflammatory cytokines. Mind-body interventions targeting depressive symptoms, physical activity, and pain-related coping may promote healthy physical and emotional functioning and improve KOA biomarkers in these patients. We adapted a mind-body and activity program for live video delivery tailored to patients with this comorbidity (GetActive-OA). This study assessed the program’s initial feasibility, credibility, and acceptability, as well as preliminary signals of improvement in pain, multimodal physical function, emotional function, coping, and KOA biomarker outcomes. Consistent with the early stages of the NIH model of intervention development, this study was not powered or intended to evaluate program efficacy. Rather, it aims to refine study procedures (eg, screening, recruitment, and assessment), identify participants’ perceptions of the study or intervention (eg, acceptability, credibility, and barriers and facilitators to participation), and iteratively refine the program in preparation for a resource-intensive, fully powered RCT.

The phase 1 focus groups revealed the unique challenges, limitations of previous medical treatments, interest and expected benefits of a mind-body activity program for KOA, barriers and facilitators to participation, and minor concerns about data collection (eg, travel to clinics and blood draws). These findings provided important information about the unique needs of patients with KOA, depression, and obesity, which we used to tailor GetActive-OA and study procedures to reduce reported barriers to adherence (eg, group-based intervention for accountability, structured goal setting, teaching skills to tolerate increased walking or lifestyle changes, and regular reminders for walking or home practice) and increasing walking (eg, address misconceptions that walking is harmful for individuals with KOA). On the basis of these findings, clinicians and researchers working with this population should consider assessing functioning across physical, emotional, and social domains. Focus group participants openly described experiences of stigma and highlighted the importance of patient-provider relationships. To build trust and encourage participation in treatment and studies, clinicians and researchers should take the time to understand participants’ underlying challenges and validate their efforts before introducing possible changes to weight, exercise, or depression.

The benchmarks for the phase 2 open pilot study indicated that the GetActive-OA program and study procedures met the criteria on nearly all a priori benchmarks for feasibility, acceptability, expectancy, and satisfaction. The exit interview findings complemented our feasibility marker results, as participants had favorable views of the live video delivery, group structure, and mind-body and activity skills. The group identified several external barriers to participation, which, coupled with concerns about the time commitment (100% of declined enrollments), suggest a need to shorten the program to optimize feasibility. The subsequent RCT will increase program reinforcements to further increase adherence, such as individualized support from study staff, recordings on the program website, and reminders for attendance and home practice. We plan to de-emphasize the skills that either participants did not use (adaptive thinking) or did not meaningfully reduce negative thoughts and emotions related to pain and depression. To improve the healthy eating component, we will streamline dietary education to bolster in-session meditation exercises (eg, mindful eating and urge surfing), with a focus on modifying their relationship with eating.

The phase 2 quantitative results offer preliminary evidence that GetActive-OA is sensitive to population-specific needs and that the measures are sensitive to changes in key outcomes [98,99]. We observed small to moderate improvements in KOA-related pain, KOA symptoms, and physical functioning and large improvements in general coping, arthritis self-efficacy, pain catastrophizing, depression, and anxiety on the quantitative outcomes. We observed minimal changes in quality of life, pain intensity, and disability, perhaps because of the high KOA severity endorsed by participants. During the exit interviews, some participants reported that their walking *increased* with the help of mind-body skills (eg, mindful walking) and linking walking to personally meaningful activities. This aligns with support from the literature that deep breathing and mindfulness can facilitate physical activity goals and decrease depression, obesity, and pain in individuals with OA [34-36]. Given this pattern of results, the lack of perceived improvement on the MPGIC was surprising. Negative perceptions on the MPGIC may be due to several factors, such as unrealistically high initial expectations for improvement, realization of the effort required to develop healthier habits during the program, and regret about how much they engaged with the program. In the subsequent RCT, study staff and clinicians will help participants set more realistic goals for the program based on their ability levels and interests. It will also provide an opportunity to further explore the correlations between perceptions of improvement and KOA outcomes in a larger sample.

In addition to changes in self-reported knee function and psychosocial factors, pre- and posttest changes in biological markers were also observed, including large improvements in the inflammatory marker IL-6 and small improvements in TLR4. IL-1β values were often below the limits of detection, suggesting that this biomarker might not be viable for assessing systemic changes in the fully powered RCT. In contrast, we observed large increases in biomarkers of cartilage breakdown (CTX-II). Although increased CTX-II levels have predicted subsequent KOA progression [93], it remains unclear whether CTX-II acutely increases as a direct result of increased physical activity. Increasing physical activity may trigger a short-term increase in cartilage turnover; however, this may be offset by attendant cartilage synthesis. As such, the subsequent RCT will assess the relative ratio of cartilage catabolism and synthesis by measuring CTX-II as well as CS846, which is a biomarker of cartilage synthesis [104].

### Limitations

Our study has several limitations. First, although we successfully recruited a complex medical population in underserved rural areas with limited access to quality care, our sample comprised exclusively women. This may have been due to several factors, including the risk of sampling bias in small samples, higher prevalence of both KOA and depression among women than among men in the United States, and stigma surrounding depression and other mental health problems [1,105]. Only patients who reported depressive symptoms and were willing to learn skills to better manage these symptoms in a group setting were included in this study. The data suggest that men and individuals from rural areas have higher levels of internalized mental health or depression stigma (ie, the tendency to agree with and internalize negative stereotypes that apply to oneself) [106,107] and thus may have been unwilling to participate. When screening and enrolling patients for the subsequent RCT, we will focus on normalizing stress and negative thoughts or emotions when living with KOA and obesity for men, specifically, while also avoiding terms such as *psychological disorder* or *major depression* to minimize stigma and increase acceptability for all patients.

Although GetActive-OA and study procedures met most of the a priori feasibility benchmarks, poor adherence to ActiGraph and blood collection at the postintervention time point hindered the interpretability of signals of improvement and should be addressed for the subsequent RCT. All participants wore the ActiGraph at baseline, but only 1 participant logged sufficient valid wear time at the postintervention time point to analyze pre- and posttest changes. Although we assessed familiarity with technology during the focus groups, we did not formally assess the technology profile of individual participants, which limits insight into specific factors that influenced adherence with either the live video aspects of the intervention delivery or activity monitoring performed at the pre- and posttest phases. Informal feedback collected by the study clinicians and research coordinators suggests low participant buy-in with ActiGraph. We will boost ActiGraph adherence for the subsequent RCT through improved incentive structures (eg, structuring compensation in proportion to the number of days and hours of wear time), explaining the purpose of the devices (eg, establishing a step count baseline for individualized quota-based pacing), and clarifying misconceptions raised during the focus groups (eg, no location tracking). We will also consider technological strategies to increase adherence, such as notification on wearable devices that they are not being worn and visualizing or gamifying step count data. In addition, owing to 4 unsuccessful blood draws, only 1 participant had both baseline and postintervention data. This was likely due to two factors: (1) a trained clinical research coordinator who completed a phlebotomy course collected blood specimens at baseline rather than expert phlebotomists and (2) consistent with the focus group results, blood draws can be more challenging in patients with obesity [108]. For the subsequent RCT, we will ensure that trained phlebotomists collect all blood samples to reduce participant burden and prevent missing data.

### Conclusions

This study depicts the development and preliminary feasibility of the first mind-body and activity program (GetActive-OA) for patients with KOA, depression, and obesity—prevalent and challenging-to-treat comorbidities for which effective treatment is lacking. Participants’ needs, preferences, and responses to the initial program yielded valuable suggestions for clinicians and researchers seeking to better support this patient population and integrate mind-body and activity interventions as complementary approaches for KOA, depression, and obesity. On the basis of our mixed methods results, we will refine the program to increase feasibility for participants (6 sessions, 45 minutes) and more comprehensively target all 3 comorbidities (renamed as *GetHealthy-OA*). The next phase will involve a live video pilot RCT (N=60 participants) of the GetHealthy-OA program with a time- and attention-matched health enhancement control (Health Enhancement Program) [68,109]. The study will yield critical information on how participants might engage differently with the intervention and control, as well as definitive information on feasibility, acceptability, and signal of improvement in the intervention before investment of resources in a fully powered efficacy trial.

## Appendix

Multimedia Appendix 1 Focus group quotes that illustrate themes and subthemes.

## Abbreviations

ASES: Arthritis Self-Efficacy Scale
IL-1β: interleukin 1-beta
IL-6: interleukin 6
KOA: knee osteoarthritis
KOOS: Knee Injury and Osteoarthritis Outcome Score
MCID: minimum clinically important difference
MGH: Massachusetts General Hospital
MOCS-A: Measure of Current Status–Part A
MPGIC: Modified Patient Global Impression of Change
NIH: National Institutes of Health
NRS: Numerical Rating Scale
OA: osteoarthritis
PASIPD: Physical Activity Scale for Individuals With Physical Disabilities
PCS: Pain Catastrophizing Scale
PHQ-9: 9-item Patient Health Questionnaire
PROMIS: Patient-Reported Outcomes Measurement Information System
RCT: randomized controlled trial
REDCap: Research Electronic Data Capture
TLR4: Toll-like receptor 4
UK: University of Kentucky
