# Supplementary material for: Live Video Mind-Body Program for Patients With Knee Osteoarthritis, Comorbid Depression, and Obesity: Development and Feasibility Pilot Study
Source: JMIR Form Res. 2022 Apr 27;6(4):e34654. doi: 10.2196/34654 (PMC9096632; doi:10.2196/34654)
Supplement: Multimedia Appendix 1 [file formative_v6i4e34654_app1.docx]

**Multimedia Appendix 1**

**Table S1.** Focus group quotes that illustrate themes and subthemes.

| Theme and subtheme | | Quotes |  |
| --- | --- | --- | --- |
| **Living with comorbid KOA^a^, depression, and obesity** | | | |
|  | Challenges | - “It’s hard to get out of bed and go do things when you know it’s going to be uncomfortable, depressing and just not make you feel any better even though you accomplished things. You do it at a price.” - “The problem that I’m having with my knees because it swells, and it causes me pain, and I have some weakness. My concern is—is it just my knees, or is it all of my joints?” |  |
|  | Causes | - “I mean the arthritis of course as you grow older the joints wear out. Having the excess weight doesn’t help it either because you’re putting more pressure on your joints and on your bones.” |  |
|  | Connection among the 3 conditions | - “It’s kind of a vicious cycle. You get depressed because you don’t feel like exercising, but you feel like exercise will help your depression and it bites its own head. It’s really tough and the arthritis is just like I’m ready to do something about my weight and I want to exercise, but when you start exercising, everything hurts. And you can only take so many painkillers...So, your hands are tied in more ways than one and it’s very frustrating and depressing.” - “I think the depression feeds the weight gain which makes the arthritis worse. You gain weight because you’re depressed and don’t want to exercise, and the extra weight and the depression make the arthritis worse.” |  |
| **Traditional and complementary treatments of KOA, depression, and unhealthy weight** | | |  |
|  | Medical treatments for all 3 conditions and outcomes (helpful/not helpful) | - “It’s the band aid approach. You’re treating the symptoms without treating the disease. Yes, it may help, but in the long-term, the disease is going to progress. You can’t stop it.” - “I was told in the very beginning that I wasn’t a candidate for surgery, so it doesn’t seem like that’s an option for me and I think that’s because of my weight.” - “I have prescribed medication for depression. I take it when I feel like I need to take it. I know that’s probably not exactly how I’m supposed to take it, but it makes me groggy, and so I don’t take a full dose all the time.” |  |
|  | Communication with doctors | - “I haven’t had a whole lot of conversation about [depression and weight], so that’s I guess that’s probably why I wanted to go ahead and get involved in this group.” - “You go to the doctor and the first thing they are looking at is your weight...Especially if it’s a doctor that you don’t know well. They don’t care about [the arthritis], everything is dependent on ‘just lose weight and you’ll be great.’ I want to get the care that I need but I can’t.” |  |
|  | Other treatments | - “I did a little bit of physical therapy, but the biggest problem was finding the time and working the expense in my budget.” - “I haven’t really tried any other interventions and the depression is part of that. I don’t really want to fight it any longer. You get to a point where you’ve been struggling and fighting, and you get tired of the fight.” |  |
|  | How to manage KOA, depression, and unhealthy weight | - “Without motivation, it’s really hard to focus. If you don’t have someone to push you or don’t have a specific goal or even if you do, when you’re depressed, it’s very hard to get motivated to do anything on your own.” - “I think if I could control my depression better, I would have more energy and it would be easier to be motivated to exercise. It would increase my strength and flexibility and lose weight, thereby removing the extra pressure from my knees.” |  |
| **Perceptions of increasing walking** | | |  |
|  | Difficulty starting/sustaining walking | - “Uneven pavement is my enemy, anything uneven. Just a rock or a walnut or something like that will cause me to fall.” - “Other things getting in the way...delaying yourself from doing it, wanting to procrastinate. Because you always put somebody else above yourself.” |  |
|  | Facilitators to starting/sustaining walking | - “I like to be out in the fresh air and see different scenery. - “Setting small goals, small accomplishable goals instead of trying to go, *‘*I’m going to walk 45 minutes today and I’m going to...’*—*like, you don’t want to set yourself up for that. And then if you don’t make it, it’s like, ‘Oh, I failed let me go eat some of those cookies,’ you know?” |  |
| **Perception of program** | | |  |
|  | Intervention components | - “What I like too is that it’s a mind and body approach. It’s not one or the other. They have to work together to make everything work.” (positive impression) - “I know that this can happen where a client feels that their nutrition part is being crammed down their throat, to where they get discouraged enough to like, ‘Forget this I’m leaving this alone.’ In other words, stress the importance of it, but don’t make it because I know I’ve dealt with in the past where I felt like I was getting a lecture.” - “I don’t like meditation. I don’t like journaling. I don’t—so, I mean, some people like it, some people swear by it. But, I mean, you can put it in there as an option, you know what I mean? And whatever works for the individual, let it work for them, but I’m just not into meditation and stuff myself.” (negative impression) |  |
|  | Barriers/facilitators to participating | - “If from week to week, you see you’re gaining something from it, then that would bring people back I think.” (facilitator) - “You get to meet new people...and we can all like bounce ideas and struggles off of one another, and it’s good to hear like when you were saying nobody’s there to judge one another, but it’s also good to hear that you’re not the only one going through the same struggle.” (facilitator) - “Either I follow the directions, or I just reap the consequences of not...There are times when I do make better choices and then there are times when like my depression will set in or something could be going on, and I don’t make good choices.” (Barrier) |  |
|  | Perception of home practice | - “[Having an app would be] Lovely. It’s a lot more convenient than trying to write it down.” - “I don’t have a problem with being asked to do—it’s, ‘Am I going to? Am I gonna wanna do it? Am I’m gonna have the energy to do it?’” - “Setting aside 10 minutes a day for your well-being—your overall well-being. That’s not too much to ask.” |  |
| **Perceptions of data collection** | | |  |
|  | Blood/urine | - “You gotta find someone who has a good stick...I have rolling veins.” |  |
|  | ActiGraph | - “I think it wouldn’t be that difficult. As long as it fits around me, I’d have no problem.” |  |
|  | Self-report | - “Me doing it online I guess would be easier.” |  |

^a^KOA: knee osteoarthritis.
